# Draft genome sequence of *Xanthomonas fragariae* reveals reductive evolution and distinct virulence-related gene content

**DOI:** 10.1186/1471-2164-14-829

**Published:** 2013-11-25

**Authors:** Joachim Vandroemme, Bart Cottyn, Steve Baeyen, Paul De Vos, Martine Maes

**Affiliations:** Institute for Agricultural and Fisheries Research (ILVO), Plant Sciences Unit - Crop Protection, Merelbeke, Belgium; Laboratory of Microbiology, Ghent University, K. L. Ledeganckstraat 35, Ghent, 9000 Belgium

## Abstract

**Background:**

*Xanthomonas fragariae* (Xf) is a bacterial strawberry pathogen and an A2 quarantine organism on strawberry planting stock in the EU. It is taxonomically and metabolically distinct within the genus *Xanthomonas*, and known for its host specificity. As part of a broader pathogenicity study, the genome of a Belgian, virulent Xf strain (LMG 25863) was assembled to draft status and examined for its pathogenicity related gene content.

**Results:**

The Xf draft genome (4.2 Mb) was considerably smaller than most known *Xanthomonas* genomes (~5 Mb). Only half of the genes coding for *TonB*-dependent transporters and cell-wall degrading enzymes that are typically present in other *Xanthomonas* genomes, were found in Xf. Other missing genes/regions with a possible impact on its plant-host interaction were: i) the three loci for xylan degradation and metabolism, ii) a locus coding for a ß-ketoadipate phenolics catabolism pathway, iii) *xcs*, one of two Type II Secretion System coding regions in *Xanthomonas*, and iv) the genes coding for the glyoxylate shunt pathway. Conversely, the Xf genome revealed a high content of externally derived DNA and several uncommon, possibly virulence-related features: a Type VI Secretion System, a second Type IV Secretion System and a distinct Type III Secretion System effector repertoire comprised of multiple rare effectors and several putative new ones.

**Conclusions:**

The draft genome sequence of LMG 25863 confirms the distinct phylogenetic position of Xf within the genus *Xanthomonas* and reveals a patchwork of both lost and newly acquired genomic features. These features may help explain the specific, mostly endophytic association of Xf with the strawberry plant.

**Electronic supplementary material:**

The online version of this article (doi:10.1186/1471-2164-14-829) contains supplementary material, which is available to authorized users.

## Background

*Xanthomonas fragariae* (Xf) is a bacterial strawberry pathogen and the cause of angular leaf spot. It was first described in the United States in 1962 [1] and has since spread globally. Under favourable conditions the pathogen may cause significant damage to both plant stock and strawberry production [2]. Xf is a quarantine pest on planting stock within the EU [3], which may explain why this generally considered mild pathogen has remained at the heart of scientific and legislative debate for decades. Xf is a distinct and homogeneous species within the otherwise complex and highly dynamic genus *Xanthomonas*[4–7]. A certain degree of infraspecific diversity within Xf has been observed, but in general it is considered as a coherent and stable species [8–10].

Unlike its clear taxonomic position, the disease-related capabilities of Xf are still obscure. One well-established characteristic of Xf is its narrow host range: *Fragaria* spp. are the only natural hosts, although close relatives of *Fragaria,* such as *Potentilla fruticosa* and *P. glandulosa*, showed symptoms after artificial inoculation and therefore are considered potential hosts [11]. Another, poorly characterized feature of Xf is its symptomless persistence in strawberry crops [12], which holds significant relevance for Xf as quarantine organism in strawberry planting stock. Molecular testing repeatedly demonstrated Xf presence in symptomless rhizomes of strawberry plants intended for planting [13, 14]. Knowledge on the *in planta* movement of Xf is limited:so far, only one study presented experimental evidence for the endophytic spread of Xf down from infected strawberry leaves to the rhizome and to newly emerging runners and daughter plants [15]. Xf is a challenging organism to study because of its fastidious nature on most common growth media [1], and its rapidly declining viability after contact with strawberry leaf extracts [16]. Moreover, Xf appeared insusceptible to genetic manipulation, which hampered our efforts in developing fluorescent and functional mutants (unpublished results).

A whole genome sequence of Xf can provide insight in its life style and help solve some of the technical problems it presents in the laboratory. Recent advances in sequencing technology and bioinformatics, together with emerging commercial whole genome sequencing services, have resulted in rapid and cost-effective means of generating draft genomes fit for most plant-pathology related studies [17]. Also within *Xanthomonas*, multiple genome sequences are available and already provided interesting insights in the most common pathogenicity determinants of the genus [18, 19]. One of the final technical challenges associated with next-generation sequencing techniques is the presence of repetitive genomic sequences [20]. Multiple paired-read datasets with varying insert sizes are often used to resolve assembly ambiguities associated with these repetitive sequences, or at least to bridge sequence gaps by concatenating related contigs into larger scaffolds. In addition, several software tools providing automatic scaffold gap-closure have recently been released: Gapcloser [21], IMAGE [22] and Gapfiller [23].

The aim of the current study was to generate a draft genome sequence of a Belgian, virulent Xf strain (LMG 25863) and to analyse its virulence-related gene content by comparison to available *Xanthomonas* whole-genome sequences. Two commercially obtained paired-read datasets were combined, and an automatic gap-closure algorithm was applied, to overcome encountered assembly problems related to repetitive DNA. Here, we present the resulting draft genome sequence of Xf LMG 25863 and the observed virulence-related features.

## Results and discussion

### Repetitive DNA content complicates genome assembly

Assembling the draft genome sequence of Xf was more challenging than anticipated. A first *de novo* assembly using a single Paired-End (PE) Illumina short-read dataset (Table 1) did not meet our expectations: although the contig number and N50-values of this initial draft genome sequence were on par with comparable assemblies of other *Xanthomonas* genomes (e.g. [24]), it was considerably smaller than anticipated (3,9 Mb instead of 5 Mb) and revealed an exceptionally high Insertion Sequences (IS) related repetitive DNA content. A second, Mate-Paired (MP) Illumina short-read dataset with a larger insert size was generated to avoid incomplete genome assembly caused by read ambiguity. The MP dataset did not improve the *de novo* assembly (data not shown), and the initial PE-based *de novo* assembly was used to start a second assembly stage that included scaffolding of the *de novo* assembly with the MP dataset, and subsequent application of the automatic gap-closing algorithm Gapcloser [21]. There was a clear improvement in both assembly quality and read disambiguation, and the current draft sufficed for our plant pathology aimed research goals. Given the quick evolution of genome assembly algorithms, the public available raw sequence data generated in this study could result in a finished genome assembly in the near future.

**Table 1:** **Main characteristics of initial** ***de novo*** **and final draft genome assembly of** ***X. fragariae*** **LMG 25863**

	***de novo***assembly	Final draft genome sequence
**Contigs (> 200 bp)**	478	96
**Total Contig Size (bp)**	3.877.791	4.182.545
**N50 contig number** ^**a**^	61	10
**N50 length (bp)** ^**b**^	21.221	131.420
**Average coverage**	158	159
**Mapped Reads (% of total)**	12.846.936 (88.8%)	14.345.704 (99.2%)
**Reads in Aligned Pairs (% of total)**	8.485.028 (58.7%)	11.731.234 (81.1%)

^a^minimum set of contigs that represent at least 50% of total genome sequence. ^b^Size of the smallest contig in the N50 set.

**Figure 1:**
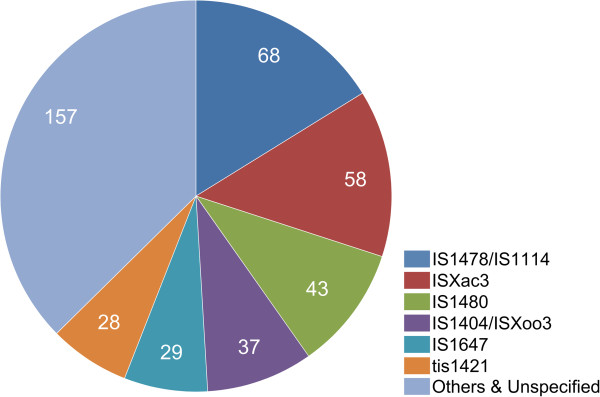
**Insertion sequences (IS) families in the genome of** ***X. fragariae*** **LMG 25863.** Overview of the most abundant Insertion Sequences (IS) families in the genome of *X. fragariae*, as annotated by the RAST online annotation pipeline.

In its current form, the draft genome of LMG 25863 consists of 96 contigs with a total contig size of 4.182.545 bp. The final draft genome of LMG 25863 was confirmed to contain an abundant IS-content. During RAST annotation, for example, 420 of the total 3786 recognized Coding DNA Sequences (CDS) in the draft genome were identified as IS-related and represented 5% of the total genome size. Of course, this number may be artificially inflated by partial and frame-shifted ORFs caused by incomplete assembly of the highly repetitive IS. However, the frequent association of the IS with sequence gaps, ambiguous read positions and orphan contigs in the draft genome all seem to confirm their abundance. Truncated CDS, due to incomplete assembly in the Xf draft genome or in the other 25 available *Xanthomonas* genomes (Table 2), were assumed as complete in our comparative analysis. The frequently encountered IS-families in Xf seemed common for *Xanthomonas* genomes (Figure 1), although during blast queries two types annotated in RAST as “IS1647” and “tis1421” appeared to be more related to *Ralstonia* and *Burkholderia* genomes.

**Table 2:** **Genomes used in this study**

Strain	Host	Disease	Genome size (Mb)	Genbank record^b^	Reference
*X. albilineans* GPE PC73	Sugarcane	Leaf Scald	3.76	[Genbank:NC_013722]	[25]
*X. arboricola* LMG 19145	Strawberry	Leaf Blight^a^	4.86	unreleased	Unpublished
*X. arboricola* LMG 19146	Strawberry	Leaf Blight^a^	5.09	unreleased	Unpublished
*X. arboricola* pv. *pruni* LMG 25862	Apricot, Plum & peach	Bacterial Spot	5.02	unreleased	Unpublished
X. *axonopodis* (citri) pv. *citri* 306	Citrus	Bacterial Canker A	5.17	[Genbank:NC_003919]	[26]
*X. axonopodis* pv. *citrumelonis* F1 (FL 1195)	Citrumelo	Bacterial Spot	4.97	[Genbank:NC_016010]	[27]
*X. axonopodis* pv. *punicae* LMG 859	Pomegranate	Bacterial Blight	4.94	[Genbank:NZ_CAGJ01000000]	[28]
*X. campestris* (*vasicola*) pv. *musacearum* NCPPB 4381	Banana	Bacterial Wilt	5.42	[Genbank:NZ_ACHS01000000]	[29]
*X. campestris* (*vasicola*) pv. *vasculorum* NCPPB 702	Sugarcane	Gumming Disease	4.78	[Genbank:NZ_ACHT00000000]	[29]
*X. campestris* pv. *campestris* ATCC 33913	Crucifers	Black Rot	5.08	[Genbank:NC_003902]	[26]
*X. campestris* pv. *campestris* 8004	Crucifers	Black Rot	5.15	[Genbank:NC_007086]	[30]
*X. campestris* pv. *campestris* B100	Crucifers	Black Rot	5.08	[Genbank:NC_010688]	[31]
*X. campestris* pv. *raphani* 756C	Crucifers	Leaf Spot	4.94	[Genbank:NC_017271]	[32]
*X. campestris* pv. *vesicatoria* (*X. euvesicatoria*) 85-10	Pepper & tomato	Bacterial Spot A	5.18	[Genbank:NC_007508]	[33]
*X. citri* pv. *mangiferaeindicae* LMG 941	Mango	Bacterial Canker	5.11	[Genbank:NZ_CAHO00000000]	[34]
*X. fragariae* LMG 25863	Strawberry	Angular Leaf Spot	4.18	[Genbank:AJRZ00000000]	This study
*X. fuscans* pv. *aurantifolii* ICPB 11122	Citrus	Bacterial Canker B	4.88	[Genbank:NZ_ACPX00000000]	[35]
*X. fuscans* pv. *aurantifolii* ICPB 10535	Citrus	Bacterial Canker C	5.01	[Genbank:NZ_ACPY00000000]	[35]
*X. gardneri* ATCC 19865	Pepper & tomato	Bacterial Spot D	5.53	[Genbank:NZ_AEQX00000000]	[24]
*X. oryzae* pv. *oryzae* KACC 10331	Rice	Bacterial Blight	4.94	[Genbank:NC_006834]	[36]
*X. oryzae* pv. *oryzae* MAFF 311018	Rice	Bacterial Blight	4.94	[Genbank:NC_007705]	[37]
*X. oryzae* pv. *oryzae* PXO 99A	Rice	Bacterial Blight	5.24	[Genbank:NC_010717]	[38]
*X. oryzae* pv. *oryzicola* BLS256	Rice	Leaf Streak	4.83	[Genbank:NC_017267]	[32]
*X. perforans* 91-118	Tomato	Bacterial Spot C	5.26	[Genbank:NZ_AEQW00000000]	[24]
*X. sacchari* NCPPB 4393	Sugarcane, insects		4.9	[Genbank:NZ_AGDB00000000]	[29]
*X. vesicatoria* ATCC 35937	Pepper & tomato	Bacterial Spot B	5.53	[Genbank:NZ_AEQV00000000]	[24]

Alphabetic overview of the 26 *Xanthomonas* strains whose genomes were compared in this study, together with their associated natural hosts and diseases, genome size, Genbank records and relevant literature references, when available. Where the current consensus classification of the organism deviates from the given name in Genbank, the current consensus classification is given between brackets. ^a^Disputed in Vandroemme et al. [39]. ^b^Only the Genbank records of the chromosomes are given.

### Phylogenetic affiliation of *X. fragariae* to other *Xanthomonas* species

**Figure 2:**
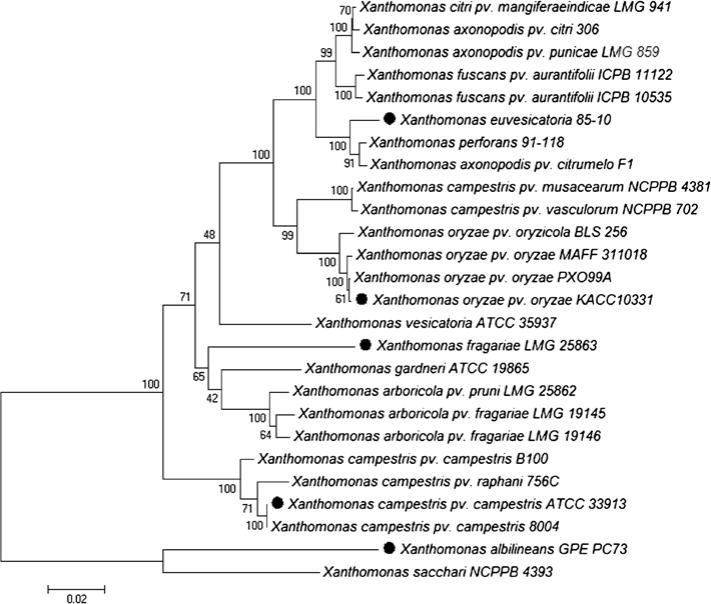
**Relationship among 26** ***Xanthomonas*** **genomes.** Phylogenetic relationship among the 26 available *Xanthomonas* genomes based on the concatenation of partial gene sequences of *gyrB*, *rpoD*, *atpD*, *dnaK* and *fyuA* (in total about 3788 nucleotides per strain). The tree was generated with Mega 5 software using the Neighbour Joining algorithm with 1,000 bootstrap replicates. Bootstrap support for the groups is represented on the tree at the different nodes. Branch length is proportional to divergence, the 0.02 scale represents 2% difference. The 5 genomes used in the EDGAR comparative genome analysis are indicated by black circles (●).

In this study, we compared the Xf draft genome with available whole genome sequences of 25 other *Xanthomonas* strains (Table 2). First, we determined the phylogenetic affiliation among these 26 genomes using the concatenated partial sequences of four housekeeping genes (*gyrB*, *atpD*, *dnaK*, and *rpoD*) and the structural gene *fyuA*[40–42]. The results of the MLSA (Figure 2) were congruent with earlier phylogenetic studies of the genus *Xanthomonas*[40, 41]. The subdivision between the “core” of the genus and the two outliers *X. albilineans* GPE PC73 and *X. sacchari* NCPPB 4393 was most evident. Also, Xf clearly represented a distinct phylogenetic lineage within the *Xanthomonas* core group.

### Xf reveals genome reduction similar to *X. oryzae* and *X. albilineans*

**Figure 3:**
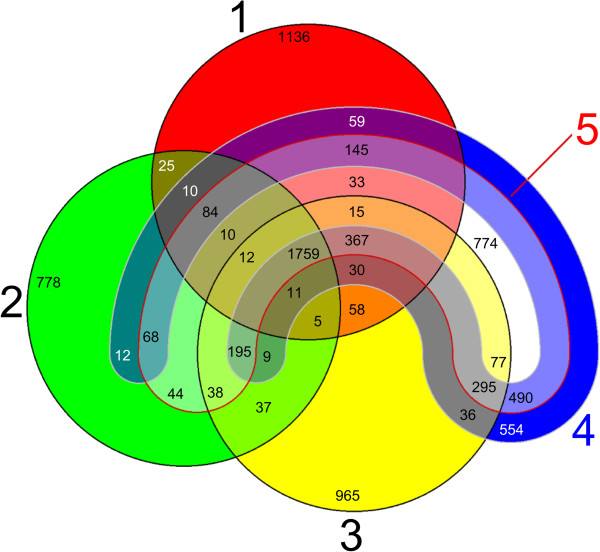
**5-way genome comparison in EDGAR.** Five-set Venn diagram constructed using EDGAR and visualizing the common gene pools among the genomes of **1)** *X. fragariae* LMG 25863, **2)** *X. albilineans* GPE PC73, **3)** *X. oryzae* pv. *oryzae* KACC 10331, **4)** *X. campestris* pv. *campestris* ATCC 33913 and **5)** *X. euvesicatoria* 85**–**10.

Even after the second genome assembly, the total contig size of the Xf draft genome (4.2 Mb) was still implying a considerable genome reduction. Because genome reduction had already been reported for *X. albilineans* (Xalb) and *X. oryzae* pv. *oryzae* (Xoo) [25], we compared the CDS content of Xf with that of Xalb GPE PC73 and Xoo KACC 10331 using the EDGAR software framework [43]. We further included *X. campestris* pv. *campestris* ATCC 33913 (Xcc) and *X. euvesicatoria* 85–10 (Xcv), respectively a vascular and a non-vascular pathogen, as references of non-reduced genomes. EDGAR analysis indicated a substantial genome reduction in Xf, which was at least in part similar to Xoo and Xalb (Figure 3). This was most apparent from the 490 CDS shared by Xcc and Xcv but absent in Xalb, Xoo and Xf. Additionally, the 195 CDS exclusively missing in Xf suggested that the genome reduction in Xf was more extensive than in Xoo (84 exclusively missing CDS), but not as extreme as in Xalb (367 exclusively missing CDS). The lists of missing and present CDS, acquired from the EDGAR analysis (Additional file 1: Table S1), were used as a starting point for all further genome comparisons in this study, and were checked by independent protein and nucleotide blast queries in all 26 *Xanthomonas* genomes before inclusion in this manuscript.

### The reduced Xf genome has the major virulence-related gene regions

The lists of CDS from the EDGAR analysis (Additional file 1: Table S1) indicated that Xf did not lose critical pathogenesis-related gene clusters reported for *Xanthomonas*. Contrary to the more reduced Xalb genome, for example, the Xf genome contained the *hrp* gene cluster coding for the structural elements of the Type III Secretion System (T3SS) and the *gum* gene cluster for extracellular polysaccharide (EPS) synthesis (Table 3). It also contained the gene cluster coding for a common Type IV Secretion System (T4SS) in *Xanthomonas,* which was entirely missing in Xoo and only partly retained in Xcv. Xf did lose the *xcs-*coded Type II Secretion System (T2SS), similar to Xoo and Xalb, but this gene cluster was reported as less critical for *Xanthomonas* pathogenicity than the clearly present *xps-*coded T2SS [44]. Also reported as unessential for virulence and absent in Xf, are the *gum*-associated genes *gumN*, *gumO* and *gumP*, and the *rpf*-associated genes *rpfD* (truncated) and *rpfI*[45].

**Table 3:** **Major virulence related gene regions in the 5** ***Xanthomonas*** **genomes compared in the EDGAR analysis**

Gene cluster	Xf	Xcc	Xcv	Xoo	Xalb
*gum* (*gumB*/*gumM*)	O1K_14575/O1K_14520	XCC2454/XCC2443	XCV2787/XCV2776	XOO3179/XOO3168	Absent
*hrp* (*hpa2*/*hpaB*)	O1K_02671/O1K_02566	XCC1241/XCC1220	XCV0441/XCV0411	XOO0096/XOO0075	Absent
LPS (*metB*/*etfA*)	O1K_19071/O1K_18996	XCC0598/XCC0619	XCV3725/XCV3711	XOO0778/XOO0790	XALc_2712/XALc_2699
*rpf* (*rpfA*/*rpfG*)	O1K_08102/O1K_08072	XCC1860/XCC1854	XCV1924/XCV1917	XOO2865/XOO2871	XALc_1349/XALc_1345
*xcs* (*xcsC*/*xcsN*)	Absent	XCC3416/XCC3426	XCV0755/XCV0765	Absent	Absent
*xps* (*xpsE*/*xpsD*)	O1K_16416/O1K_16366	XCC0660/XCC0670	XCV3658/XCV3668	XOO0847/XOO0857	XALc_2654/XALc_2664
T4SS (*virD4*/*virB6*)	O1K_12781/O1K_12736^a^	XCC2483/XCC2474^b^	XCV2810/XCV2807^c^	Absent	XALc_1842/XALc_1835^a,b^
Flagellum (*fliA*/*flgM*)	O1K_18068/O1K_17833	XCC1906/XCC1955	XCV1977/XCV2036	XOO2621/XOO2836	XALc_1379/XALc_1431

i) the *gum* gene cluster for extracellular polysaccharide synthesis, ii) the *hrp* gene cluster for Type III secretion, iii) the gene cluster for lipopolysaccharide (LPS) synthesis, iv) the *rpf* gene cluster for regulation of pathogenicity, the Type II Secretion System coding gene clusters v) *xcs* and vi) *xps*, vii) the gene cluster for the most common Type IV Secretion System in *Xanthomonas* and viii) the flagellar biosynthesis genes. Gene clusters retrieved in each genome are labeled with the locus tags of their respective first and last genes (names given between parentheses). ^a^ *virD4* absent in this gene region, but replaced by divergent homolog elsewhere in the genome, ^b^ *virB5* and *virB6* missing, ^c^all genes except *virD4*, *virB8* and *virB9* missing.

Other noteworthy gene regions that were indicated as absent in Xf by EDGAR analysis and confirmed by blast queries, were i) the glyoxylate shunt pathway coding locus [46, 47], which was also missing in Xoo and Xalb, ii) the three loci of the Carbohydrate Utilization (CUT) system involving *TonB*-dependent transporters for xylan degradation and metabolism in *X. campestris*[48, 49], iii) the genes coding for the *kdp* potassium transport system [50] and, iv) a gene region coding for a ß-ketoadipate phenolics degradation pathway [51] that was also found partly absent in Xoo (Table 4).

**Table 4:** **Noteworthy gene-regions missing in** ***X. fragariae*** **LMG 25863**

Gene cluster	Xf	Xcc	Xcv	Xoo	Xalb
Glyoxylate shunt	Absent	XCC0232/XCC0240	XCV0257/XCV0267	Absent	Absent
Phenolics Degradation I	Absent	XCC0354/XCC0363	XCV0367/XCV0377	Absent	XALc_3034/XALc_3040
Phenolics Degradation II	Absent	XCC0366/XCC0373	XCV0380/XCV0387	XOO0481/XOO0488	XALc_3021/XALc_3031
Potassium Transport	O1K_06922^a^	XCC0702/XCC0706	XCV0808/XCV0812	XOO3842/XOO3846	XALc_2828/XALc_2832
Xylan degradation I	Absent	XCC2826/XCC2828	XCV3145/XCV3147	XOO1260/XOO1262	XALc_3147/XALc_3149
Xylan degradation III	Absent	XCC4102/XCC4107	XCV4333/XCV4338	XOO4419/XOO4424	XALc_0057/XALc_0062
Xylan degradation III	Absent	XCC4117/XCC4122	XCV4357/XCV4364	XOO4427/XOO4433	XALc_0035/XALc_0040

Gene regions found absent in the genome of *X. fragariae* LMG 25863, together with their occurrence in the complete genomic sequences of 4 other *Xanthomonas* species compared in EDGAR: i) the glyoxylate shunt pathway genes, ii) the genes coding for a phenolics degradation pathway, iii) the structural genes of a potassium transporter and iv) the three loci for xylan degradation. Gene regions retrieved from each genome are labeled with the locus tags of their respective first and last genes. Absent or incomplete gene clusters are marked in grey. ^a^5′-truncated homolog of *kdpA* [Genbank:NP_636094].

Although some absent gene-regions in Xf may have virulence-related implications, the genome reduction in Xf seems to weaken nutritional and adaptive flexibility rather than clear virulence functions. For example, the absence of all three xylan degradation loci and the ß-ketoadipate pathway may indicate that Xf is unable to respectively degrade xylan and metabolize the phenolic components of lignin, two important elements of the secondary plant cell wall [49]. Perhaps, the opinion that primarily soil bacteria have been associated with lignin degradation [52], might suggest that the main role of the ß-ketoadipate pathway lays in saprophytic survival. Likewise, though the glyoxylate shunt pathway has been linked to successful symbiotic and pathogenic plant-bacterial interactions, it does so by increasing metabolic fitness through growth on C_2_-compounds [46]. Potassium is another important nutritional element, crucial for cell turgor maintenance, activation of cellular enzymes and pH homeostasis. The *kdp* potassium transport system is widely distributed among bacteria and serves as an emergency K^+^-scavenging system that is only expressed and activated under extreme environmental stress [50]. Maybe these missing functions are redundant for Xf’s existence in the strawberry leaf apoplast. Among the 26 analysed *Xanthomonas* genomes, the xylan degradation pathway, the *sdk* potassium transport system and the entire phenolics degradation pathway were uniquely missing in Xf.

### The Xf genome has a reduced *TonB*-dependent transporter set

**Figure 4:**
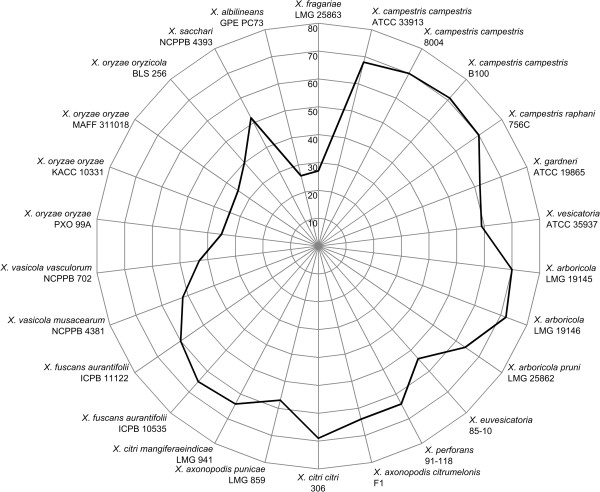
**Number of complete TBDT homologs.** Radar chart showing the number of complete *TonB*-Dependent Transporter (TBDT) homologs found in the 26 studied *Xanthomonas* genomes. Each spoke in the chart represents one strain, with a higher homolog content resulting in a more outward position of the graph on the spoke. The concentric circles form a ruler with a primary unit of 10, as indicated on the left of the spoke representing *X. fragariae* LMG 25863.

The EDGAR analysis suggested a substantial loss of *TonB*-dependent transporters (TBDT) in Xf, Xoo and Xalb. Therefore, we screened all 26 *Xanthomonas* genomes for homologs of 100 TBDT references (Additional file 2: Table S2). Overall, the average TBDT gene repertoire amounted to 56 homologs with a standard deviation of 14. Then again only 27 homologs could be found in Xf, which was the second smallest TBDT repertoire after Xalb (26 homologs) (Figure 4). The Xoo and Xoc genomes had 34 to 40 homologs. The extensive TBDT repertoire in Xcc has been linked to niche diversity and carbohydrate scavenging in the oligotrophic conditions encountered during epiphytic survival [48]. In turn, the small TBDT sets in Xf, Xalb and the *X. oryzae* strains may be yet another adaptation to a stable and restricted niche.

### Xf has a reduced plant cell wall degrading enzyme set

**Figure 5:**
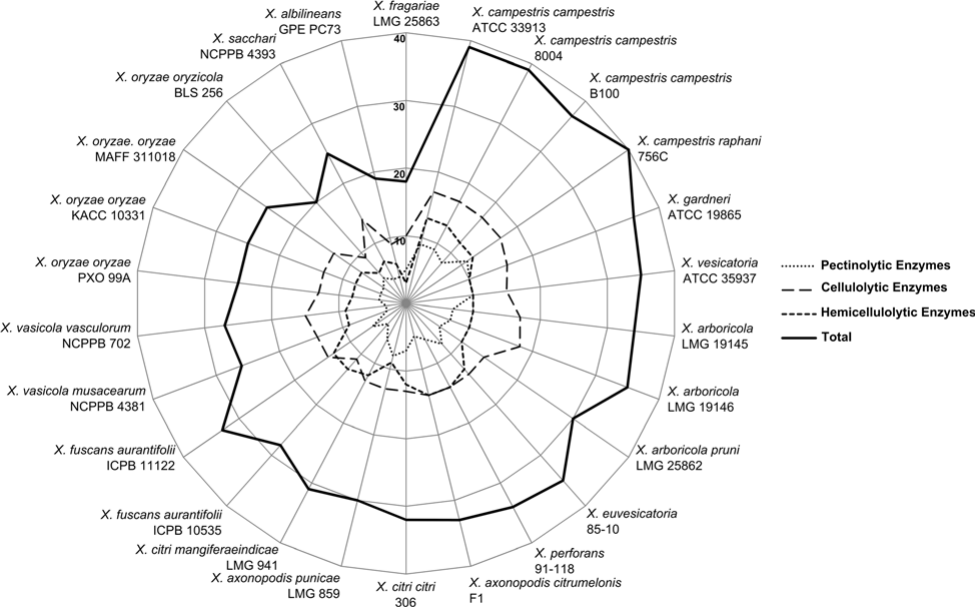
**Number of complete macerating enzymes.** Radar chart showing the number of complete homologs of pectinolytic, cellulolytic and hemicellulolytic enzymes found in the 26 studied *Xanthomonas* genomes. Each spoke in the chart represents one strain, with a higher homolog content resulting in a more outward position of the graph on the spoke. The concentric circles form a ruler with a primary unit of 10, as indicated on the left of the spoke representing *X. fragariae* LMG 25863.

Observing the missing xylan degradation and ß-ketoadipate pathways in Xf, triggered us to study its Cell Wall Degrading Enzyme (CWDE) repertoire in greater detail. To this end, all 26 *Xanthomonas* genomes were screened for homologs of 46 CWDE references (Additional file 3: Table S3). The average number of pectinolytic, cellulolytic and hemicellulolytic enzyme homologs thus retrieved in each genome was 6, 14 and 10 respectively, with standard deviations of 2, 2 and 3. The average total CWDE repertoire consisted of 31 homologs, with a standard deviation of 6. With only 18 homologs, Xf revealed the smallest CWDE repertoire comprised of an apparently unreduced pectinolytic enzyme repertoire but with low numbers of cellulolytic (10 homologs) and hemicellulolytic (3 homologs) enzymes (Figure 5). Small CWDE repertoires were again also apparent in the genomes of Xalb (19 homologs), Xoc (20 homologs), and to a lesser extent in the three Xoo strains (25 homologs). For the 26 *Xanthomonas* genomes analysed, no clear correlation could be observed between the CWDE repertoires and their infection mode: only the XCC3534-like cellobiosidase appeared unique for vascular pathogens.

Despite the contribution of CWDEs to virulence, the reduced set of Xf does not necessarily make it a lesser pathogen. Smaller CWDE repertoires are typically found in biotrophic pathogens, who rely on precise breaching of the host cell wall during infection instead of extensive tissue destruction observed for necrotrophic pathogens [53]. One potential explanation for the reduced CWDE repertoire of Xf may be found in the concurrent absence of the ß-ketoadipate phenolics degradation pathway: strawberry plant tissue is rich in phenolics [54] and many contribute to plant defence as phytoanticipins or phytoalexins, which are often released when plant cell integrity is compromised [55]. Therefore, one could hypothesize that one way for Xf to survive long-term residence in its potentially toxic host is to avoid extensive tissue damage.

### Xf exhibits a distinct T3SE repertoire and several putatively new effectors

**Figure 6:**
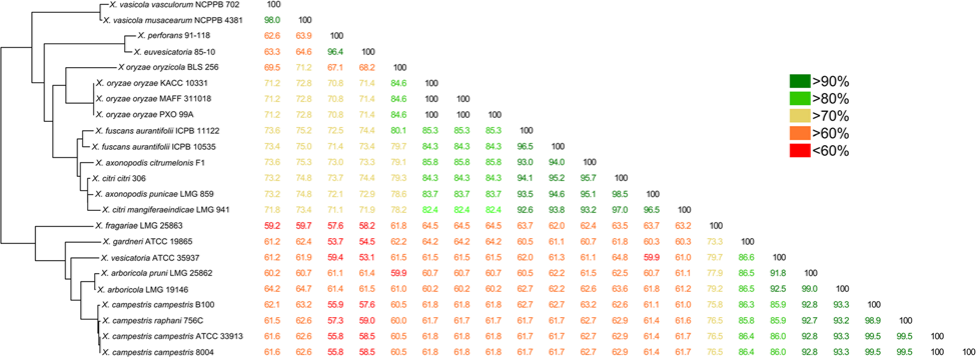
**Pairwise similarity among** ***XopA*** **homologs.** Matrix showing the pairwise protein sequence similarity between all *XopA* homologs retrieved from the 23 *hrp*-positive *Xanthomonas* genomes used in this study. The similarities between each *XopA* pair are given as per cent values and are colored according to their percentile rank as indicated in the legend. The matrix rows have been ordered in accordance with the position of each sequence in a Neighbor Joining tree based on the similarity matrix, which is shown left of the matrix. The tree was rooted manually and was not subjected to bootstrap resampling.

**Figure 7:**
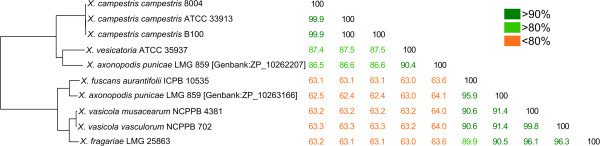
**Pairwise similarity among** ***XopAG*** **homologs.** Matrix showing the pairwise protein sequence similarity between all *XopAG* homologs retrieved from the 23 *hrp*-positive *Xanthomonas* genomes used in this study. The similarities between each *XopAG* pair are given as per cent values and are colored according to their percentile rank as indicated in the legend. The matrix rows have been ordered in accordance with the position of each sequence in a Neighbor Joining tree based on the similarity matrix, which is shown left of the matrix. The tree was rooted manually and was not subjected to bootstrap resampling. The locus tags of both *XopAG* homologs retrieved in the genome sequence of *X. axonopodis* pv. *punicae* LMG 859 are given between rectangular brackets.

Because several Xf-exclusive CDS in the EDGAR analysis showed similarity with Type III Secretion Effector (T3SE) genes, we compared the T3SE genes of Xf with those of the other 25 *Xanthomonas* genomes. Classifying the Xf-effectors within the currently defined effector-families [56], however, was challenging. Some effector families contained clearly distinct subgroups with lower than 70% inter-subgroup pairwise protein sequence similarities, as indicated here for XopA (Figure 6). A more restrictive definition and further subdivision of the current T3SE-families could improve the classification. For now, we retained the classification as defined by White et al. (2009) [56], except for *XopAG*, and applied a general cut-off of 60% pairwise protein sequence similarity within a given effector family. For XopAG we suggest the division into two subfamilies: *XopAG1* and *XopAG2*, with [Genbank:ZP_10262207] and [Genbank:ZP_10263166] as respective reference sequences, encountered in the genome of *X. axonopodis* pv. *punicae* LMG 859. The Xf-effector gene [Genbank:O1K_00020] was unambiguously classified in *XopAG2* (Figure 7). Using the 60% cut-off rule, we identified homologs of 23 known *Xanthomonas* T3SEs in Xf, with two homologs for *XopX* (Table 5). Presence of at least one *XopAD* homolog and multiple *XopP* homologs was suggested, but could not be confirmed due to incomplete genome assembly.

**Table 5:** **Known type III secretion effectors of** ***X. fragariae*** **LMG 25863**

Effector	Locus tag	Best match for Xf- sequence	Worst match among other sequences
*AvrBs2*	[Genbank:O1K_12410]	92.3%	72.5%
*Hpa2/HpaH*	[Genbank:O1K_02671]	87.6%	79.0%
*HpaA*	[Genbank:O1K_02586]	84.0%	68.2%
*XopA/hpa1*	[Genbank:O1K_02666]	79.7%	53.1%
*XopB*	[Genbank:O1K_11187]	84.6%	88.5%
*XopC1*	[Genbank:O1K_14874]	97.4%	99.9%
*XopD*	[Genbank:O1K_16938]	67.7%	78.2%
*XopE4*	Contig19 (17160..18326)	97.6%	100.0%
*XopF1*	[Genbank:O1K_02551]	82.6%	75.3%
*XopF2*	[Genbank:O1K_06487]	63.1%	55.7%
*XopK*	[Genbank:O1K_18791]	85.6%	72.0%
*XopL*	[Genbank:O1K_16566]	66.1%	60.7%
*XopN*	[Genbank:O1K_10027]	87.3%	73.4%
*XopP* ^a^	[Genbank:O1K_11182, Genbank:O1K_11197, Genbank:O1K_11202, Genbank:O1K_11207, Genbank:O1K_11212]; Contig64; Contig67		
*XopQ*	[Genbank:O1K_15074]	87.1%	71.4%
*XopR*	[Genbank:O1K_01999]	75.7%	57.4%
*XopV*	[Genbank:O1K_05602]	77.5%	71.6%
*XopX*	[Genbank:O1K_04266]	83.3%	68.1%
*XopX* ^b^	[Genbank:O1K_04271, Genbank:O1K_04281]	85.1%	68.1%
*XopZ2*	[Genbank:O1K_02556]	87.3%	88.5%
*XopAD* ^a^	[Genbank:O1K_14315]; Contig24 (1..5097); Contig57; Contig92 (89708..96529)		
*XopAE*	[Genbank:O1K_08322]	69.6%	84.7%
*XopAF*	[Genbank:O1K_18806]	72.5%	50.4%
*XopAG2*	[Genbank:O1K_00020]	96.3%	90.6%
*XopAM*	[Genbank:O1K_07544]	87.3%	80.5%
*XopAS* ^c^	[Genbank:O1K_11585]	ND	ND

Overview of known type III secretion effector homologs retrieved in *X. fragariae* LMG 25863, together with their locus tags in the whole-genome sequences. Incomplete or un-annotated loci are marked with the label of the coding contig and coding nucleotide range, when applicable. As an indication of the diversity within each effector class, the pairwise protein sequence similarity between the *X. fragariae* homolog and its best match found among the other *Xanthomonas* genomes is given, and the worst sequence match among all remaining effector sequences. ^a^Partial sequences because of incomplete genome assembly, ^b^O1K_04281 is a suspected incorrect double of O1K_04271 caused by erroneous sequence assembly, ^c^no other complete homologs in *Xanthomonas*. ND, not determined.

**Figure 8:**
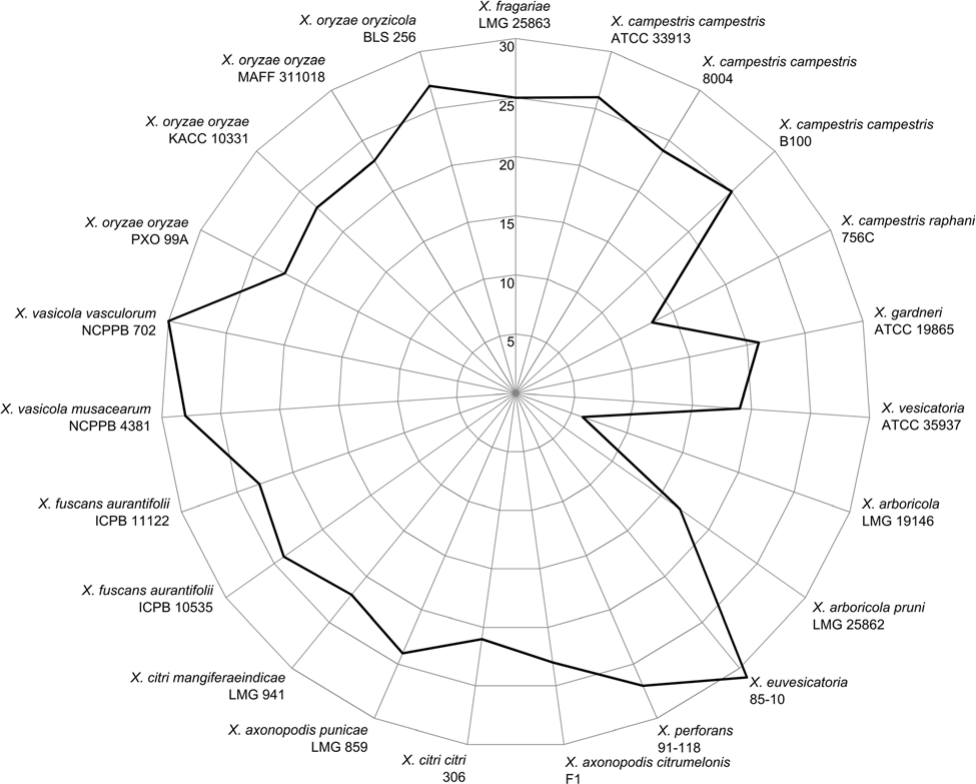
**Number of complete type III secretion effectors.** Radar chart showing the total number of represented type III secretion effector families in each of the 23 *hrp*-positive *Xanthomonas* genomes compared in this study. Each spoke in the chart represents one strain, with a higher number of represented effector families in a more outward position of the graph on the spoke. The concentric circles form a ruler with a primary unit of 5, as indicated on the left of the spoke representing *X. fragariae* LMG 25863.

Comparison of the effector gene repertoire identified in Xf and in the other 25 *Xanthomonas* genomes is shown (Additional file 4: Table S4). For simplicity, multiple homologs of a certain effector class within a single genome were marked only once. Transcription Activator-Like Effectors (TALEs) were not included in the comparison because Xf did not appear to contain any, and also TALEs are distinct from the other effectors in both coding sequences and function [57]. Among the 23 *hrp*-positive genomes, the average T3SE repertoire consisted of 23 different families, with a standard deviation of 5 (Figure 8). Explicit small T3SE repertoires were observed in *X. campestris* pv. *raphani* 756C (13 effectors) and in *X. arboricola* LMG 19146 (6 effectors) [39], while Xcv revealed the largest repertoire with 31 effectors.

**Figure 9:**
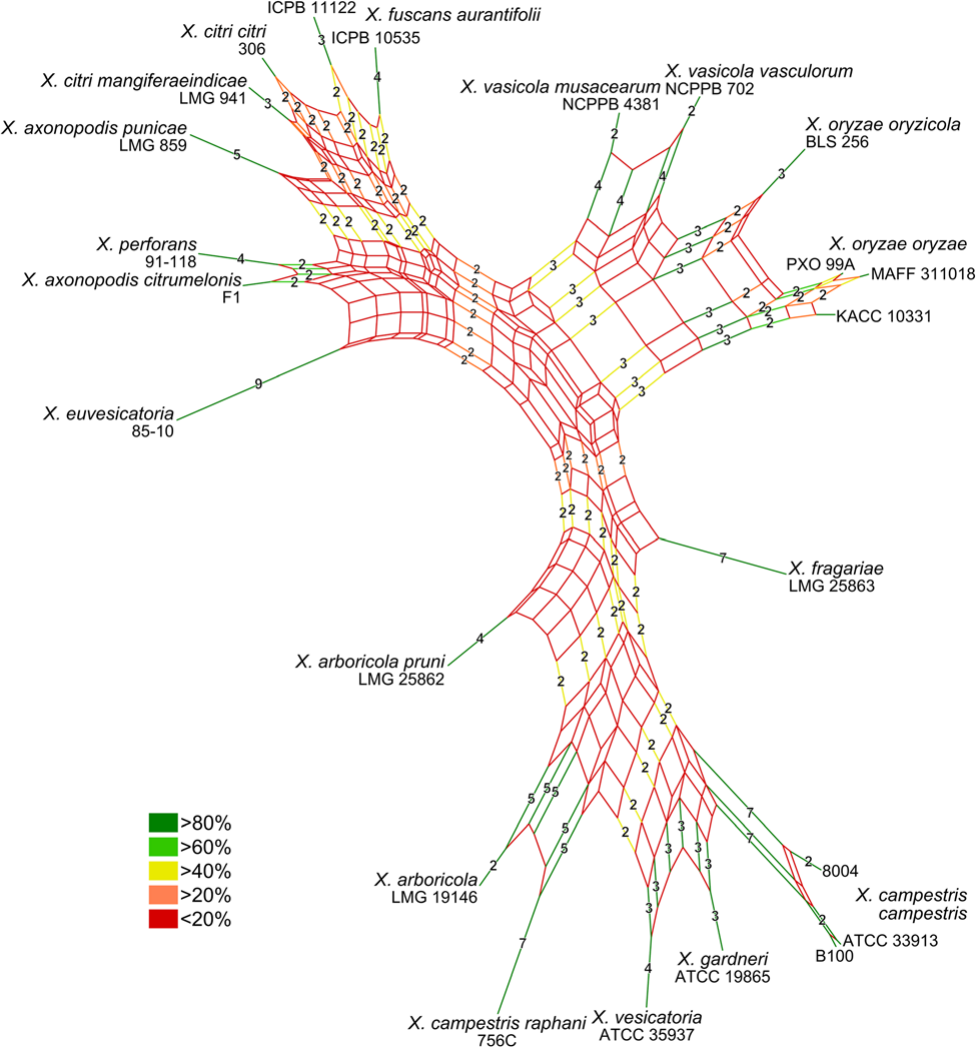
**Relationship among type III secretion effector repertoires.** Neighbor Joining split network based on binary representations of the type III effector repertoires found in the 23 *hrp*-positive *Xanthomonas* genomes compared in this study. The network visualizes all conflicting and incompatible bipartitions (“splits”) encountered during a bootstrap resampling with 1000 replicates, and each split is colour-coded according to its bootstrap support as indicated in the legend. The split-lengths were calculated using the Hamming distance and represent the number of effector flips (from absent to present or reverse) between each point in the network. Distances greater than 1 were indicated on splits with more than 40% bootstrap support. The network visualizes 94 out of 543 calculated splits or 88.1% of the total split weight, and is supported by a global cophenetic correlation of 82%.

Although the T3SE repertoire of Xf seemed average-sized (25 effectors), it distinguished itself by the presence of multiple rare effectors: XopB, XopC1, XopE4, XopZ2, XopAF and XopAS. This distinct T3SE repertoire in Xf was also evident from the isolated position of Xf in a Neighbor Joining split network based on binary representations of the T3SE repertoires found among all 23 *hrp*-positive genomes (Figure 9). Interestingly, the general topology of the T3SE-based network correlated well with the MLSA based phylogenetic tree shown before, suggesting that at least a fraction of the T3SE repertoire was acquired early and evolved slowly during the formation of the major lineages within the genus.

In addition to this unique “known” T3SE repertoire, the Xf genome also revealed several putative new effectors (Table 6). Three putative T3SEs in Xf showed low homology with the known *Xanthomonas* effectors XopC, XopE and XopAD: [Genbank:O1K_18811] showed highest pairwise similarity (66.6%) with a T3SE found in the genus *Ralstonia*, [Genbank:01K_20482] showed 56.4% similarity to an unidentified protein in *Mesorhizobium*, and [Genbank:O1K_11082] showed up to 78.5% pairwise similarity with an unidentified protein in *X. gardneri* ATCC 19865. Nine other putative T3SEs in Xf showed distant homology with both XopD and PsvA, a T3SE family in *Acidovorax* and *Pseudomonas* contributing to host specificity, plant defence suppression and *in planta* pathogen proliferation [58]. Although the function of these putative new T3SEs still needs to be confirmed, the likely Plant-Inducible-Promoter (PIP) sequences [59], frequently found in the vicinity of their coding sequences, suggest virulence-related functions for these proteins. The low%GC value observed in 7 of these coding sequences might suggest that they were acquired through horizontal gene transfer (Table 6).

**Table 6:** **Potential new type III secretion effectors found in** ***X. fragariae*** **LMG 25863**

General characteristics					Most closely related known***Xanthomonas***T3SE			Best genbank match		
Locus	Moved start-codon compared to PGAAP annotation	Protein length	%GC	Putative PIP	Effector class	Best match for Xf	Worst match amongst the sequences within the effector class	Locus	Organism	Similarity with Xf-sequence
[Genbank:O1K_18811]		424 AA	52		*XopC2*	52.8%	94.4%	[Genbank:CCA89217]	*Ralstonia*	66.6%
[Genbank:O1K_11082]	147 bp to 5*'* side	327 AA	64	ttcgcaaacgcgtgcatgctgttggc-(N)_323_-TTG	*XopE3*	55.4%	97.8%	[Genbank:ZP_08184849]	*X. gardneri*	78.5%
[Genbank:O1K_20482]	492 bp to 5*'* side	2578 AA	64	ttggcggccacgtgccgacgttgcc-(N)_244_-ATG	*XopAD*	53.4%	86.0%	[Genbank:ZP_09295361]	*Mesorhizobium*	56.4%
[Genbank:O1K_01564]	507 bp to 5*'* side	570 AA	56	ttcggcaaacccactacgccttcgc-(N)_222_-ATG	*XopD*	46.9%	78.2%	[Genbank:EGH27374]	*Pseudomonas*	55.7%
[Genbank:O1K_01569, Genbank: O1K_01579]^a^	975 bp to 5*'* side	580 AA	56	ttcggcaaacccactacgccttcgc-(N)_222_-ATG	*XopD*	47.8%	78.2%	[Genbank:EGH27374]	*Pseudomonas*	54.7%
[Genbank:O1K_01589]	399 bp to 5*'* side	583 AA	57	ttcggcaaagccgctacgccttcgc-(N)_206_-ATG	*XopD*	47.9%	78.2%	[Genbank:EGH27374]	*Pseudomonas*	55.1%
[Genbank:O1K_02164]	453 bp to 5*'* side	666 AA	56	ttcggcaagcccgctacgccttcgc-(N)_193_-ATG	*XopD*	49.3%	78.2%	[Genbank:EGH27374]	*Pseudomonas*	53.2%
[Genbank:O1K_03146]	633 bp to 5*'* side	860 AA	57	ttcggcaaacctgctacgccttcgc-(N)_156_-ATG	*XopD*	52.0%	78.2%	[Genbank:EGH27374]	*Pseudomonas*	47.8%
[Genbank:O1K_03991]	342 bp to 5*'* side	584 AA	55		*XopD*	47.9%	78.2%	[Genbank:EGH27374]	*Pseudomonas*	54.0%
[Genbank:O1K_13828]	891 bp to 5*'* side	675 AA	55	ttcggcaaacccgctacgccttcgc-(N)_210_-ATG	*XopD*	47.5%	78.2%	[Genbank:EGH27374]	*Pseudomonas*	51.6%
[Genbank:O1K_20327]	246 bp to 5*'* side	619 AA	56	ttcggcaaacccgttacgccttcgc-(N)_224_-ATG	*XopD*	47.9%	78.2%	[Genbank:EGH27374]	*Pseudomonas*	55.8%

Overview of possible new type III secretion effectors in *X. fragariae* LMG 25863 together with their Genbank accessions, protein length in amino acids, %GC-content of the coding DNA sequences, and possible Plant-Inducible-Promoter (PIP) boxes. Putative start-codons of most open reading frames were moved from the start as identified by PGAAP as indicated in the table. The pairwise protein sequence similarity between each new Xf-effector and the most closely related *Xanthomonas* effector class is compared with the lowest pairwise similarity observed within this effector class. Finally, the accession number of the best protein blast match in Genbank for each putative new Xf-effector is given together with the organism it comes from, and its pairwise protein sequence similarity with the Xf-effector. ^a^O1K_01579 is a possible incorrect double of O1K_01569, created by incorrect sequence assembly.

### Xf harbours a Type VI secretion system similar to *X. oryzae*

**Figure 10:**
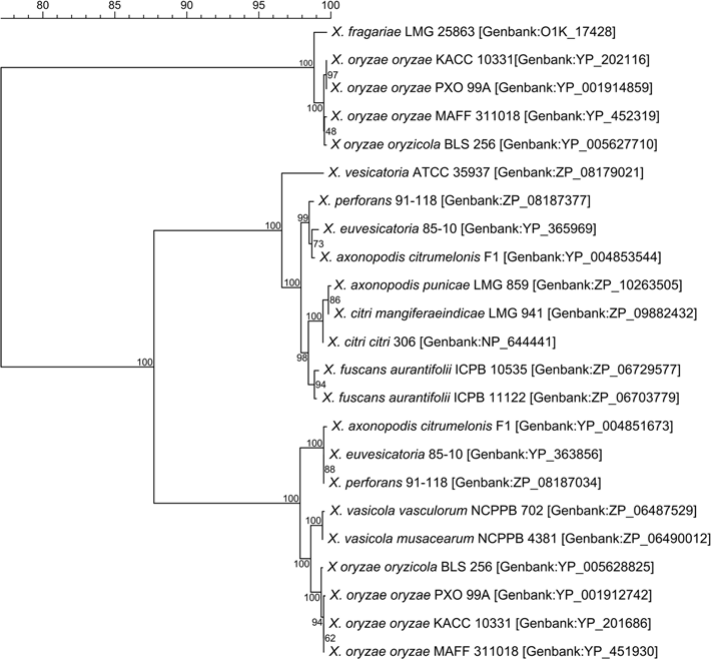
**Relationship among type VI secretion systems.** Relationship among the type VI secretion system-related COG3519-family proteins retrieved from the 26 *Xanthomonas* genomes compared in this study. The tree was constructed using the Neighbor Joining method and was rooted manually. Global sequence similarities are shown as per cent values in the axis and bootstrap values calculated with 1000 replicates are shown on the cluster nodes. Genbank accession numbers are given between rectangular brackets.

Two loci shared between Xf and Xoo in the EDGAR analysis appeared to code for structural elements of a Type VI secretion system (T6SS). Coding sequences for putative Vgr protein-like T6SS effectors were encountered in the Xf genome, although their exact number and sequence could not be established due to incomplete assembly. The T6SS is the most recently found secretion system in gram-negative bacteria, and several distinct types are widely distributed among the *Proteobacteria*[60]. In contrast to their common structure, which is analogous to the injection apparatus of bacteriophages, the specific roles of each of these distinct T6SSs are still obscure: some systems were shown to contribute to the modification of eukaryotic hosts within both pathogenic and symbiotic relationships, while others were linked to inter-bacterial activity during the struggle for niche dominance [61, 62]. All 26 *Xanthomonas* genomes were searched for the presence of T6SSs using a widely retained T6SS-related protein class (COG3519) as bait [63], which revealed the presence of three distinct T6SSs within the genus (Figure 10). The T6SS of Xf appeared highly similar to one of the two T6SSs that were found in the *X. oryzae* genomes. This distinct, previously thought *X. oryzae*-exclusive, T6SS has been correlated with plant host specialization because of its similarity with the T6SS found in plant pathogens like *Ralstonia solanacearum* and *Pseudomonas syringae*[60]. The same study grouped the other two T6SSs that were observed here, in a widely distributed T6SS class present in both animal and plant pathogens, suggesting a broader functionality. Of course, this in-silico based hypothesis would need further experimental confirmation.

### Evidence of considerable horizontal gene transfer and a CRISPR in Xf

In addition to some virulence genes that are possibly acquired by Horizontal Gene Transfer (HGT) and IS elements, other evidence of HGT-exposure could be found in the Xf genome. The IS-content appeared exceptionally abundant in Xf. While IS abundance is a common feature of *Xanthomonas* genomes, exceptionally high IS content was previously reported for the three Xoo strains and the Xoc strain [32, 36–38]. There, it was interpreted as a result of the consistent association of these strains with rice: the stable environment would have alleviated the selective pressure of many genes, allowing their disruption by IS. A similar process could be envisioned for Xf. Conversely, it has also been described as an important source of genome plasticity in *X. oryzae* and a cause of the genotypic diversity within the species [32, 38]. This seems to conflict with the restricted genotypic diversity reported for Xf. Perhaps the relatively young practice of formal plant breeding in strawberry cultivation (18th-19th century AD) [64] compared to the ancient domestication of rice (7000–4000 BC) [65] has to be considered.

Possible HGT-related CDS in Xf were a set of phage coding genes ([Genbank:O1K_11280] to [Genbank:O1K_11410]), nine toxin-antitoxin (TA) modules (Table 7) [66], and nine contigs with a total sequence length of 25 Kb exhibiting more than 90% overall DNA sequence identity with the 27 Kb Plasmid II of *X. albilineans* GPE PC73. The latter nine contigs mainly coded for the structural elements of a second, phylogenetic distinct T4SS, and plasmid replication functions. Although this raised the possibility of an autonomous plasmid in Xf, so far we were unable to identify or isolate any plasmid DNA from the sequenced strain (data not shown).

**Table 7:** **Toxin-antitoxin modules in** ***X. fragariae*** **LMG 25863**

Toxin	Antitoxin	Putative toxin/antitoxin-system
[Genbank:O1K_03731]	[Genbank:O1K_03726]	RelE/RelB
[Genbank:O1K_03776]	[Genbank:O1K_03771]	HigB/HigA
[Genbank:O1K_04356]	[Genbank:O1K_04361]	ParD/ParE
[Genbank:O1K_07102]	[Genbank:O1K_07097]	DinJ/YafQ
[Genbank:O1K_07172]	[Genbank:O1K_07177]	VapB/VapC
[Genbank:O1K_12891]	[Genbank:O1K_12886]	RelE/StbE
[Genbank:O1K_17078]	[Genbank:O1K_17073]	CcdB/CcdA
[Genbank:O1K_04456]	[Genbank:O1K_04461]	Phd/Doc
[Genbank:O1K_04466]	[Genbank:O1K_04471]	MazE/MazF

Genbank accession numbers of toxin and antitoxin sequences identified in *X. fragariae* LMG 25863 by Genbank protein sequence blast queries together with their putative toxin-antitoxin module family.

Another HGT-related CDS in Xf with a potential virulence related function, [Genbank:O1K_06242], coded for a 2485aa putative Repeats-in-toxin (RTX) exoprotein [67]. Its low%GC (54%) and the presence of IS elements directly up and downstream seem to indicate that it was acquired through HGT.

Finally, the Xf genome revealed a Clustered Regularly Interspaced Short Palindromic Repeats (CRISPR)-region comprised of 6 CRISPR associated (cas) genes of the so-called Ypest (“*Yersinia pestis*”) subtype ([Genbank:O1K_01919] to [Genbank:O1K_01944]), a 121 bp long AT-rich leader sequence and a CRISPR containing 36 identical repeats and 36 spacers. A second, smaller locus containing an additional 4 repeats and 3 spacers was also found, although the last repeat in this locus was degenerated. Among all other studied *Xanthomonas* genomes, cas-genes of the Ypest-subtype were also found in Xalb and *X. campestris* pv. *raphani* 756C (Xcr). Moreover, the associated CRISPR repeat sequence in Xf (GTTCACTGCCGCGTAGGCAGCTCAGAAA) was identical to that of Xcr, and diverged only one nucleotide from that of Xalb (GTTCACTGCCG**T**GTAGGCAGCTCAGAAA). CRISPR regions were recently recognized as a prokaryotic adaptive immune system against invading DNA molecules, with functional analogy to the RNA interference (RNAi) pathways in eukaryotes [68].

## Conclusion

The draft genome sequence of Xf provided valuable insight in its general and more specific pathogenicity-related gene content. Although the current total contig size of 4.2 Mb is not definite, the sheer amount of missing gene homologs in Xf is sufficient evidence for a significant genome reduction. A similar “convergent genome erosion” was already reported for *X. albilineans* and *X. oryzae* pv. *oryzae,* and ascribed to their restricted lifestyle within the xylem vessels of their hosts [25]. A similar genome reduction was found here in Xf and also in *X. oryzae* pv. *oryzicola* BLS256, which are two non-vascular leaf pathogens. Therefore, it may be more accurate to ascribe the convergent genome reduction of these *Xanthomonas* species to their endophytic lifestyle and typically to their commitment to a single host. Similar to earlier comparative genomic studies within *Xanthomonas*[30, 45], we were unable to find clear determinants for host or tissue specificity. Perhaps this specificity is the result of a more complex interplay between different genes or, of a subtle sequence variation within a small set of conserved genes. Alternatively, clear host or tissue determinants may still remain hidden within the substantial group of proteins for which we currently have no clear molecular function, or in uncharacterised functional RNAs.

Based on the data presented here, one could hypothesize an evolutionary process for Xf that is reminiscent of the model that was recently presented for some dangerous epidemic bacteria of humans [69]. During an initial period of intense horizontal gene transfer experienced by the more generalist ancestor of Xf, acquirement of certain heterologous host-specificity factors would have allowed it to colonize the strawberry leaf apoplast and thus escape antagonists and environmental threats. This transition from a dynamic to a stable environment would subsequently have triggered the observed genome reduction: useless or redundant features, especially metabolic, perceptive and regulatory functions, were allowed to degrade and eventually were lost. At some point, the progressing genome erosion resulted in the effective metabolic “entrapment” of Xf within the strawberry plant, excluding it from other hosts or more general epiphytic or saprophytic lifestyles. Because of Xf’s increasing spatial and phylogenetic isolation, the initially intense horizontal gene transfer would have abated, a process that was perhaps hastened by the acquirement of the CRISPR region. Meanwhile, mobile genetic elements which conferred a selective advantage to Xf would have become permanently incorporated in the genome. This evolutionary process would finally have resulted in the genotypical and phenotypical distinct, mainly endophytic, biotrophic and strawberry-specific Xf known today. The eventual necrosis of the typical water-soaked angular leaf spots associated with Xf would not be in conflict with this hypothesis: it could be the manifestation of an eventual breakthrough of the plant defence, or merely the collapse of an overburdened plant cellular system.

Of course, many of the in-silico based hypotheses presented here should be tested and confirmed by further experimental data. In our opinion, the most appealing matters for future research are i) the molecular and functional characterization of the putatively new *PsvA*-like T3SEs, ii) the exact function of the T6SS in Xf and other *Xanthomonas*, and iii) the delicate endophytic existence of Xf, sensitive to toxic compounds inside the strawberry plant cells.

## Methods

### Strain selection, culture conditions and DNA preparation

The selected Xf strain, LMG 25863, was isolated as GBBC-Xf 920 in 2002 from clear angular leaf spots on strawberry leaves at the Institute for Agricultural and Fisheries Research (ILVO) in Belgium. Since then, it has been applied in the development of a real-time PCR detection method for Xf [70], served as Xf reference in a study of *X. arboricola* pv. *fragariae*[39] and as parental strain in the development of a green fluorescent Xf mutant (unpublished). It was deposited at the BCCM-LMG culture collection at the time of whole genome sequencing. For genomic DNA preparation, the cryogenically stored Xf strain was resuscitated by incubation on Wilbrinck-N agar medium [71] at 28°C for 96 h. A single colony of this culture was then transferred to fresh Wilbrink-N agar plates and again incubated at 28°C for 48 h. From these cultures, a total of 30 μg DNA with a concentration of at least 100 ng/μl and an OD260:280 rating between 1.8 and 2.0 was prepared using the Gentra Puregene Cell Kit (QIAGEN Benelux B.V., Venlo, The Netherlands), according to the manufacturer’s instructions. Quantity and quality of the extracted DNA was checked using the NanoDrop 1000 spectrophotometer (Thermo Fisher Scientific, Wilmington, DE, USA).

### Sequencing, draft genome assembly and annotation

Custom DNA library preparation and Illumina-sequencing was performed by Baseclear N.V., Leiden, The Netherlands. A first Paired-End (PE) DNA library with an a mean insert size of 375 bp was sequenced with 50 bp reads on an Illumina Genome Analyzer IIx (Illumina Inc., San-Diego, USA). A second, Mate-Paired (MP) DNA library with a mean insert size of 5100 bp was sequenced with 75 bp reads on an Illumina Hiseq2000 (Illumina Inc.), but only the first 50 bp were used to avoid chimeric reads.

The received raw PE and MP read sets were quality trimmed in CLC Bio v4.0 (CLC bio, Aarhus, Denmark) using a Phred quality cut-off score of 20. An initial *de novo* assembly was performed in CLC Bio v4.0 using only the PE reads, and all contigs shorter than 200 bp were discarded. This assembly was scaffolded in SSPACE Premium v2.0 [23] using MP reads and processed with the Gapcloser v1.12 tool of the SOAP genome assembly software [21]. Gapfiller and IMAGE were not used because the former was not yet freely available and the latter could not be operated as intended. Because Gapcloser did not recognize the Hiseq2000 file parsing of the MP dataset, only the PE data was used. Finally, the draft genome sequence was manually edited with the Editseq tool of DNAStar Lasergene core suite v10.0.1 (DNASTAR Inc., Madison, WI, USA). Remaining N-nucleotides in the scaffolds, introduced during scaffolding and not replaced by gapcloser, were removed from the final sequence by breaking up the scaffolds back into contigs where they were encountered. The quality of the final draft genome sequence was compared to the initial PE-based *de novo* assembly through comparative read-mapping in CLC Bio v4.0 using both the trimmed read sets. The final draft genome sequence of Xf was putatively annotated with the RAST v4.0 online annotation pipeline [72] and NCBI’s Prokaryotic Genomes Automatic Annotation Pipeline (PGAAP; http://www.ncbi.nlm.nih.gov/genomes/static/Pipeline.html).

### Comparative genome analysis

The presence of possible pathogenicity-related genes in the Xf draft genome was analysed by comparison with 25 other *Xanthomonas* genomes (Table 2). In a first explorative screening, the gene content of the Xf genome was compared with the chromosomes *X. campestris* pv. *campestris* (Xcc) ATCC 33913, *X. euvesicatoria* (Xcv) 85–10, *X. oryzae* pv. *oryzae* (Xoo) KACC 10331 and *X. albilineans* (Xalb) GPE PC73 in EDGAR [43]. Absence or presence of coding sequences in each genome, as reported by EDGAR, were independently confirmed by performing protein and nucleotide blast queries (as described below) in the target genomes before inclusion in this manuscript.

In a second phase, gene families of interest were examined in all 26 genomes. All genomes except three were screened using the protein and nucleotide blast tools of Genbank. Four genomes not present in Genbank were screened with the blast tool of the SEED Viewer v2.0 web-interface [73]: draft genomes of the present Xf strain LMG 25863, two *X. arboricola* strains LMG 19145 and LMG 19146 [39], and the *X. arboricola* pv. *pruni* strain LMG 25864 (M Maes, unpublished). Based on the EDGAR results, three protein families were studied in greater detail: the *TonB*-dependent transporters (TBDT), the cell-wall degrading enzymes (CWDE) and the Type III secretion system effectors (T3SE).

### Phylogenetic relationship

To determine the phylogenetic position of Xf within the *Xanthomonas* genus, we performed in silico multi-locus sequence analysis using partial sequences of the genes *gyrB*, *rpoD*, *atpD*, *dnaK* and *fyuA*, according to Parkinson et al. (2009) [40], Young et al. (2008) [41], and Ngoc et al. (2010) [42]. Sequences were retrieved from the Xf and the 25 other *Xanthomonas* genomes available in GenBank (Table 2). The sizes of the five partial sequences are 530 bp (*gyrB*), 747 bp (*atpD*), 940 bp (*dnaK*), 873 bp (*rpoD*) and 698 bp (*fyuA*), giving a total of 3788 bp for the concatenated dataset. Sequences were concatenated and aligned using the CLUSTALW algorithm [74]. Sequence alignment, trimming, and phylogenetic analysis were performed in Mega5 [75]. The phylogenetic tree was generated using the Maximum Composite Likelihood method for calculating distances and the Neighbor Joining algorithm for clustering [76], bootstrap analysis was performed using 1000 bootstrap replicates.

### TonB-dependent receptors

The TBDT repertoire of Xf was compared to that of the other 25 *Xanthomonas* genomes. The 72 TBDTs identified in Xcc ATCC 33913 [48] were used as primary references. Initially, the 26 genomes were screened for homologs of these primary references using protein blast queries. In case of a negative blast result, an additional nucleotide blast with the reference coding sequence was performed to exclude false negative results. TBDTs with low protein sequence similarity (<70%) with the primary references were considered as new types and were added to the list of references. TBDTs in the more distantly related *X. albilineans* GPE PC73 and *X. sacchari* NCPPB 4393 occasionally showed ambiguous homology with more than one reference. This was resolved by reciprocal blast, after which the TBDT in question was assigned to the reference with the best blast hit score.

### Cell-wall degrading enzymes

Reference sequences of different CWDE-families were retrieved from [26]. All 26 genomes were searched for homologs of these references in a similar fashion as applied for the TBDTs.

### Type III secretion effectors

References of the presently known T3SEs in *Xanthomonas* were retrieved from the *Xanthomonas* Resource website [56] and all 26 genomes were screened for homologs. Because of their sometimes large diversity, the protein sequences of all encountered (putative) effectors were collected and compared in Bionumerics v6.6 (Applied Maths, Sint-Martens-Latem, Belgium). For each effector class, a protein-sequence based pairwise alignment similarity matrix was calculated using the standard Bionumerics algorithm in its default settings. Candidate effectors of Xf were only considered as part of an effector class when it exhibited at least 60% pairwise similarity with at least one other entry in the matrix. Next, a binary table listing the presence or absence of at least one fully coded homolog of every known T3SE class in each of the 26 genomes was created. Truncated, frame-shifted or otherwise suspected incomplete or inactive coding sequences were interpreted as absent. When the full coding sequence could not be retrieved because of incomplete assembly, the effector was counted as present. Multiple homologs of an effector in a single genome were counted only once. This binary dataset was imported and analyzed in Bionumerics. A binary-data based distance matrix was calculated using the Hamming Distance parameter and a distance-based consensus network was calculated using the Neighbor Joining tree method in its default settings and bootstrap resampling with 1000 replications [77].

### Depositions

The raw sequence data received from Baseclear N.V. (Leiden, The Netherlands) was deposited at the Short Read Archive (SRA) of Genbank under accession numbers SRR514114 (PE dataset) and SRR514113 (MP dataset). The current draft genome sequence was deposited at Genbank under accession number AJRZ00000000 after automatic annotation by the PGAAP online annotation pipeline.

## Electronic supplementary material

Additional file 1: Table S1: EDGAR output table. Raw output data of the EDGAR genome comparison, listing all CDSs shared among *X. fragariae* LMG 25863, *X. albilineans* GPE PC73, *X. oryzae* pv. *oryzae* KACC 10331, *X. campestris* pv. *campestris* ATCC 33913 and *X. euvesicatoria* 85–10. (XLSX 143 KB)

Additional file 2: Table S2: *TonB*-dependent transporters. Occurrence of *TonB*-dependent transporters among the 26 studied *Xanthomonas* genomes. The 100 reference protein sequences are listed in the table rows, with the name of the genome they were retrieved from and their Genbank locus tags indicated in the first two columns. The remaining columns show the occurrence of homologs of each reference protein among the 26 genomes. “2”: two homologs present; “1”: one homolog present, “0”: no homolog found, “ΨT”: coding DNA sequence encountered, but protein believed inactive due to truncation;” ΨF”: coding DNA sequence encountered, but protein believed inactive due to frameshift, “Seq”: coding DNA sequence truncated due to incomplete genome-assembly; functional protein assumed present during further processing of data. Underlined entries were not annotated in Genbank, and were retrieved using nucleotide blast queries. (XLSX 21 KB)

Additional file 3: Table S3: Macerating enzymes. Occurrence of macerating enzymes among the 26 studied *Xanthomonas* genomes. The 46 reference protein sequences are listed in the table rows, with the name of the genome they were retrieved from and their Genbank locus tags indicated in the second and third columns. The remaining columns show the occurrence of homologs of each reference protein among the 26 genomes. “2”: two homologs present; “1”: one homolog present, “0”: no homolog found, “ΨT”: coding DNA sequence encountered, but protein believed inactive due to truncation;” ΨF”: coding DNA sequence encountered, but protein believed inactive due to frameshift, “Seq”: coding DNA sequence truncated due to incomplete genome-assembly; functional protein assumed present during further processing of data. Underlined entries were not annotated in Genbank, and were retrieved using nucleotide blast queries. (XLSX 20 KB)

Additional file 4: Table S4: Type III secretion effectors. Overview of the type III secretion effector repertoire in each of the 26 tested *Xanthomonas* genomes. “1”: at least one homolog present, “0”: no homolog found, “ΨT”: coding DNA sequence encountered, but protein believed inactive due to truncation;“ ΨF”: coding DNA sequence encountered, but protein believed inactive due to frameshift,“ ΨIS”: coding DNA sequence encountered, but protein believed inactive due to inserted sequence, “Seq”: coding DNA sequence truncated due to incomplete genome-assembly; functional protein assumed present during further processing of data. Underlined entries were not annotated in Genbank, and were retrieved using nucleotide blast queries. (XLSX 15 KB)
